# Association of Uric Acid With Blood Pressure in Hypertension Between Treatment Group and Non-treatment Group

**DOI:** 10.3389/fcvm.2021.751089

**Published:** 2022-01-11

**Authors:** Ning Ding, Yong Long, Changluo Li, Liudang He, Yingjie Su

**Affiliations:** Department of Emergency Medicine, The Affiliated Changsha Central Hospital, Hengyang Medical School, University of South China, Changsha, China

**Keywords:** uric acid, blood pressure, hypertension, systolic blood pressure, diastolic blood pressure

## Abstract

**Objective:** This study aimed to explore the association between uric acid (UA) and blood pressure (BP) in hypertension treatment and non-treatment groups.

**Methods:** A cross-sectional study with 6,985 individuals from the National Health and Nutrition Examination Survey (NHANES) was performed. Multiple linear regression analysis was performed to explore the relationship of UA and BP in hypertension between the treatment group (*n* = 5,983) and the non-treatment group (*n* = 1,002).

**Results:** A significantly negative association was discovered in SBP (β, −0.36 [95% CI, −0.71, −0.01]) and DBP (β, −0.47 [95% CI, −0.69, −0.26]) in the hypertension treatment group. In the hypertension non-treatment group, the associations between UA and BP including SBP, DBP were both an inverted U-shape. The inflection point of SBP and DBP was 7 and 7.5 mg/dl, respectively. For SBP, the association was positively significant (β, 3.11 [95% CI, 1.67, 4.56]) before the inflection point of 7 mg/dl. However, after the inflection point of 7 mg/dl, the association was negative (β, −5.44 [95% CI, −8.6, −2.28]). For DBP, the inflection point was 7.5 mg/dl, and the effect size was positive (β, 1.19 [95% CI, 0.37, 2.01]) before the inflection point. However, after it, the effect size was negative (β, −3.24 [95% CI, −5.72, −0.76]).

**Conclusion:** The association between UA and BP was negative in the hypertension treatment group. In the hypertension non-treatment group, the associations between UA and BP including SBP and DBP were both an inverted U-shape.

## Introduction

Serum uric acid (UA) is the final product of purine metabolism, and seafood, fats, and red meat are rich in it. It has been confirmed to be associated with many diseases, such as hypertension (1), diabetes (2), heart failure (3), diabetic kidney disease (4), and cerebrocardiovascular diseases (5). Previous studies have demonstrated that elevated level of serum UA was correlated with an increase in blood pressure (BP) and the occurrence of hypertension (1, 6, 7). However, the results of clinical trials to control BP through uric acid-lowering therapy were not so convincing. One study with a total of 137 patients with hypertension and hyperuricemia who were treated with xanthine oxidase inhibitors (allopurinol or febuxostat) explored BP changes before and after uric acid-lowering treatment. No significant change in BP was identified (8). One recent meta-analysis concluded that there was still a lack of high-level evidence to support the use of uric acid-lowering therapy to improve BP control in adults with hypertension (9). Previous studies have mainly focused on the relationship between UA and BP or hypertension. However, few studies have been performed to explore the association between UA and BP in hypertension.

Hence, in order to investigate the association between UA and BP in hypertension, we conducted a cross-sectional study composed of 6,985 participants from the 2009–2018 National Health and Nutrition Examination Survey (NHANES) database. Moreover, all possible potential confounding factors were adjusted to evaluate the association between UA and BP in hypertension more accurately.

## Methods

### Study Population

**Figure 1:**
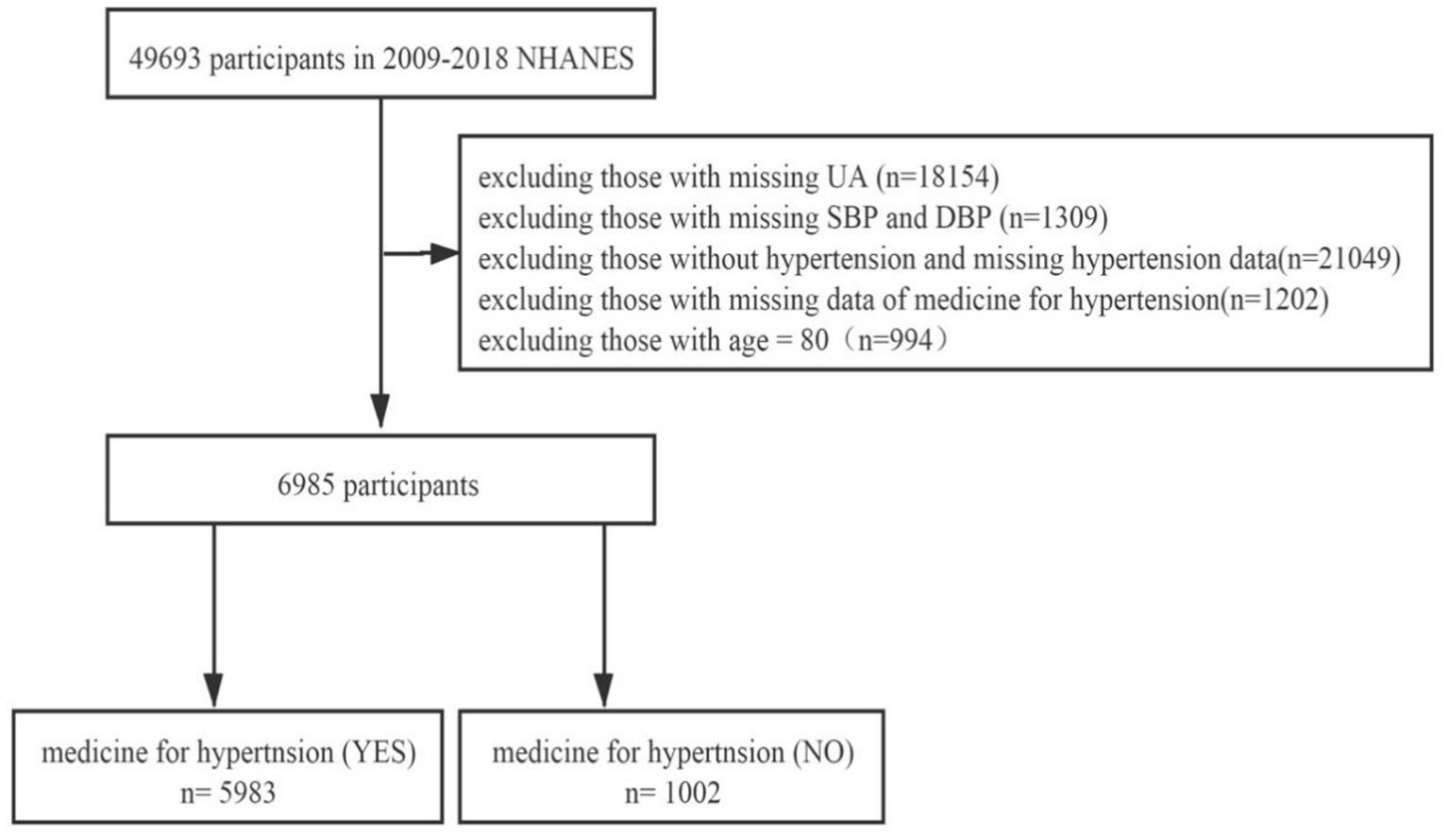
Flowchart of the study design and participants excluded from the study. UA, uric acid; SBP, systolic blood pressure; DBP, diastolic blood pressure.

We searched the NHANES database for 5 periods of data from 2009 to 2018 for research. Of the 49,693 potential participants in the study, 42,708 were excluded for the following reasons: missing UA data (*n* = 18,154), missing systolic blood pressure (SBP) and diastolic blood pressure (DBP) data (*n* = 1,309), without hypertension and missing hypertension data (*n* = 21,049), missing data of medicine for hypertension (*n* = 1,202), and age = 80 (*n* = 994). The reason why people (=80 years old) were excluded was because NHANES defined all the people (≥ 80 years old) as 80 years old. In the end, 6,985 people were included in the study, and they were divided into two groups: the hypertension treatment group (*n* = 5,983) and hypertension non-treatment group (*n* = 1,002; Figure 1).

### Baseline Measurements and Definitions

In this study, UA was the exposed variable, and BP was the primary outcome including systolic blood pressure (SBP) and diastolic blood pressure (DBP). After the participants sat down with their feet supporting the ground, they rested quietly for at least 5 min, and three blood pressure readings were obtained with a mercury sphygmomanometer. Each reading was separated by 30 s. If one or more previous readings were interrupted, the fourth reading was obtained. SBP and DBP were made up of the average of all available measurement data. Race was divided into four categories: Mexican-American, White, Black, and Other. Age was divided into four groups, which included <18, 18–44, 45–59, and ≥ 60. Body mass index (BMI) was divided into five groups, which were underweight (< 18.5 kg/m^2^), normal weight (18.5~24.99 kg/m^2^), overweight (25~29.99 kg/m^2^), obesity (≥ 30 kg/m^2^), and not recorded. Alcohol consumption was divided into three groups based on the question: “In the past 12 months, how often did you drink any type of alcoholic beverage?” The groups were composed of drinking, no drinking, and not recorded. Diabetes was divided into four groups based on the question, “Have you ever been told by a doctor or health professional that you have diabetes or sugar diabetes?”, and were yes, no, borderline, and not recorded. Smoking was divided into three groups based on the question, “Do you now smoke cigarettes?”, and were smoking, no smoking, and not recorded. Glomerular filtration rate (GFR) was estimated by the simplified Modification of Diet in Renal Disease (MDRD) equation 186 × SC^−1.154^ × Age^−0.203^× (0.742 if female) (10). The method of obtaining other variables, such as UA, creatinine (CR), glucose (GLU), hemoglobin (HGB), total cholesterol (TC), low-density lipoprotein (LDL), and high-density lipoprotein (HDL), can be found at www.cdc.gov/nchs/nhanes/.

### Statistical Analysis

The associations between UA and BP including SBP and DBP were explored by applying a multiple linear regression model. Mean ± SD (normal distribution) or median (quartile) (skew distribution) was applied to represent continuous variables, including CR, GLU, HGB (missing data *n* = 18), HDL (missing data *n* = 1), TC (missing data *n* = 1), GFR, SBP, DBP, UA, and LDL (missing data *n* = 3,634). Percentage or frequency was utilized to represent categorical variables, including gender, race, alcohol consumption (missing proportion 19.44%), diabetes (missing proportion 0.06%), smoking (missing proportion 50.15%), age, and BMI (missing proportion 1.43%). The missing data of HGB, HDL, and TC were filled in with the median, and the missing data of LDL, alcohol consumption, diabetes, smoking, and BMI were included in the data analysis separately as a group. Smooth curve fittings were used to explore the non-linear relationship between UA and SBP or DBP. We also conducted a log-likelihood ratio test of the one-line (non-segmented) model with the piecewise regression model to determine whether the threshold exists. Statistical software packages R (http://www.R-project.org) and EmpowerStats (http://www. empowerstats. com, X&Y Solutions, Inc.) were used for the data analyses. Statistical significance was based on the value of *p* < 0.05.

## Results

### Baseline Characteristics of the Participants

A total of 6,985 participants were included in our study based on the exclusion criteria. Among them, there were 5,983 people in the hypertension treatment group and 1,002 people in the hypertension non-treatment group (Table 1). In the hypertension treatment group, the percentage of men and women was 47.38 and 52.62%, respectively. With regard to ethnicity, 11.88% were Mexican-American, 37.66% were White, and 29.82% were Black. Overall, the mean (SD) values for CR, GLU, HGB, HDL, TC, GFR, SBP, DBP, UA, and LDL were 1.02 (0.72) mg/dl, 6.44 (2.76) mmol/L, 13.78 (1.52) mg/dl, 1.34 (0.43) mmol/L, 4.88 (1.1) mmol/L, 70.24 (24.73) ml/min/1.73 m^2^, 132.75 (19.15) mmHg, 71.9 (12.62) mmHg, 5.89 (1.56) mg/dl, and 2.8 (0.93) mmol/L, respectively. Among the participants, 38.79% were <60 years old, 13.27% were of normal weight, 31.14% had diabetes, 17.48% were smokers, and 56.11% were alcohol drinkers. In the hypertension non-treatment group, percentage of males and females was 51 and 49%, respectively. With regard to ethnicity, 15.47% were Mexican-American, 36.33% were White, and 26.75% were Black. Overall, the mean (SD) values for CR, GLU, HGB, HDL, TC, GFR, SBP, DBP, UA, and LDL were 0.93 (0.62) mg/dl, 6.06 (2.98) mmol/L, 14.04 (1.75) mg/dl, 1.34 (0.43) mmol/L, 5.16 (1.11) mmol/L, 80.28 (27.19) ml/min/1.73 m^2^, 135.69 (22.12) mmHg, 78.2 (13.88) mmHg, 5.54(1.44) mg/dl, and 3.11 (0.94) mmol/L, respectively. Among the participants, 71.86% were <60 years old, 16.77% were of normal weight, 14.57% had diabetes, 29.44% were smokers, and 65.47% were alcohol drinkers. The Univariate analysis for SBP and DBP is demonstrated in Supplementary Tables 1, 2.

**Table 1:** Description of 6,985 participants included in this study.

**Medicine for hypertension**	**Yes (*n =* 5,983)**	**No (*n =* 1,002)**
Creatinine (mg/dl)	1.02 ± 0.72	0.93 ± 0.62
Glucose (mmol/L)	6.44 ± 2.76	6.06 ± 2.98
Hemoglobin (g/dl)	13.78 ± 1.52	14.04 ± 1.75
HDL (mmol/L)	1.34 ± 0.43	1.34 ± 0.43
TC (mmol/L)	4.88 ± 1.10	5.16 ± 1.11
GFR (ml/min/1.73 m^2^)	70.24 ± 24.73	80.28 ± 27.19
SBP (mmHg)	132.75 ± 19.15	135.69 ± 22.12
DBP (mmHg)	71.90 ± 12.62	78.20 ± 13.88
UA (mg/dl)	5.89 ± 1.56	5.54 ± 1.44
LDL (mmol/L)	2.80 ± 0.93	3.11 ± 0.94
Gender		
Male	2,835 (47.38%)	511 (51.00%)
Female	3,148 (52.62%)	491 (49.00%)
Race		
Mexican-American	711 (11.88%)	155 (15.47%)
White	2,253 (37.66%)	364 (36.33%)
Black	1,784 (29.82%)	268 (26.75%)
Other race	1,235 (20.64%)	215 (21.46%)
Alcohol consumption		
No drinking	1,442 (24.10%)	172 (17.17%)
Drinking	3,357 (56.11%)	656 (65.47%)
Not recorded	1,184 (19.79%)	174 (17.37%)
Diabetes		
Yes	1,863 (31.14%)	146 (14.57%)
No	3,858 (64.48%)	807 (80.54%)
Borderline	260 (4.35%)	47 (4.69%)
Not recorded	2 (0.03%)	2 (0.20%)
Smoke		
Smoking	1,046 (17.48%)	295 (29.44%)
No smoking	1,915 (32.01%)	226 (22.55%)
Not recorded	3,022 (50.51%)	481 (48.00%)
Age (years)		
16–44	602 (10.06%)	366 (36.53%)
45–59	1,719 (28.73%)	354 (35.33%)
60–79	3,662 (61.21%)	282 (28.14%)
BMI (kg/m^2^)		
<18.5	35 (0.58%)	9 (0.90%)
18.5–24.9	794 (13.27%)	168 (16.77%)
25–29.9	1,815 (30.34%)	281 (28.04%)
≥30	3,262 (54.52%)	521 (52.00%)
Not recorded	77 (1.29%)	23 (2.30%)

*HDL, high-density lipoprotein; TC, total cholesterol; GFR, glomerular filtration rate; SBP, systolic blood pressure; DBP, diastolic blood pressure; UA, uric acid; LDL, low-density lipoprotein; BMI, body mass index. Mean ± SD for: creatinine, glucose, hemoglobin, HDL, TC, GFR, SBP, DBP, UA, LDL. P-value was calculated by linear regression model. % for: gender, race, alcohol consumption, diabetes, smoking, age, BMI. P-value was calculated by chi-square test*.

### Results of Multiple Linear Regression Analyses

We analyzed the independent effect of UA on SBP and DBP with three models (Table 2). In model I, in the hypertension treatment group, a significantly negative association can be found in SBP (β, −0.49 [95% CI, −0.8, −0.18]) and DBP (β, −0.29 [95% CI, −0.5, −0.09]). In the hypertension non-treatment group, a significantly positive association can be found in SBP (β, 1.6 [95% CI, 0.66, 2.55]) and DBP (β, 1.4 [95% CI, 0.81, 2]). In model II, gender, race, and age were adjusted. In the hypertension treatment group, the relationship still existed in SBP (β, −0.59 [95% CI, −0.9, −0.27]) and DBP(β, −0.48 [95% CI, −0.68, −0.28]). In the hypertension non-treatment group, the relationship also still existed in SBP (β, 1.21 [95% CI, 0.17, 2.25]) and DBP (β, 1.2 [95% CI, 0.55, 1.85]). In model III, in which all the potential confounding factors were adjusted for, the significantly negative association was discovered in SBP (β, −0.36 [95% CI, −0.71, −0.01]) and DBP (β, −0.47 [95% CI, −0.69, −0.26]) in the hypertension treatment group. In the hypertension non-treatment group, the negative relationship was found not to exist in SBP (β, 1.12 [95%CI, −0.02, 2.26]) and DBP (β, 0.52 [95% CI, −0.19, 1.22]).

**Table 2:** Result of multiple linear regression analysis between uric acid and blood pressure in hypertension treatment group and non-treatment group.

**Model**	**Medicine for hypertension (yes)**	**Medicine for hypertension (no)**
Systolic blood pressure (β, 95%CI, *P*)
Model I	−0.49, (−0.80, −0.18), 0.0019	1.60, (0.66, 2.55), 0.0010
Model II	−0.59, (−0.90, −0.27), 0.0003	1.21, (0.17, 2.25), 0.0224
Model III	−0.36, (−0.71, −0.01), 0.0417	1.12, (−0.02, 2.26), 0.0546
Diastolic blood pressure (β, 95%CI, *P*)
Model I	−0.29, (−0.50, −0.09), 0.0048	1.40, (0.81, 2.00), <0.0001
Model II	−0.48, (−0.68, −0.28), <0.0001	1.20, (0.55, 1.85), 0.0003
Model III	−0.47, (−0.69, −0.26), <0.0001	0.52, (−0.19, 1.22), 0.1498

*CI, confidence interval; HDL, high-density lipoprotein; TC, total cholesterol; GFR, glomerular filtration rate; LDL, low-density lipoprotein; BMI, body mass index. Model I adjustment: none; model II adjustment: gender, age, and race; model III adjustment: gender, age, race; creatinine, glucose, hemoglobin; HDL, TC, GFR, LDL, alcohol consumption, diabetes, smoking, and BMI*.

**Figure 2:**
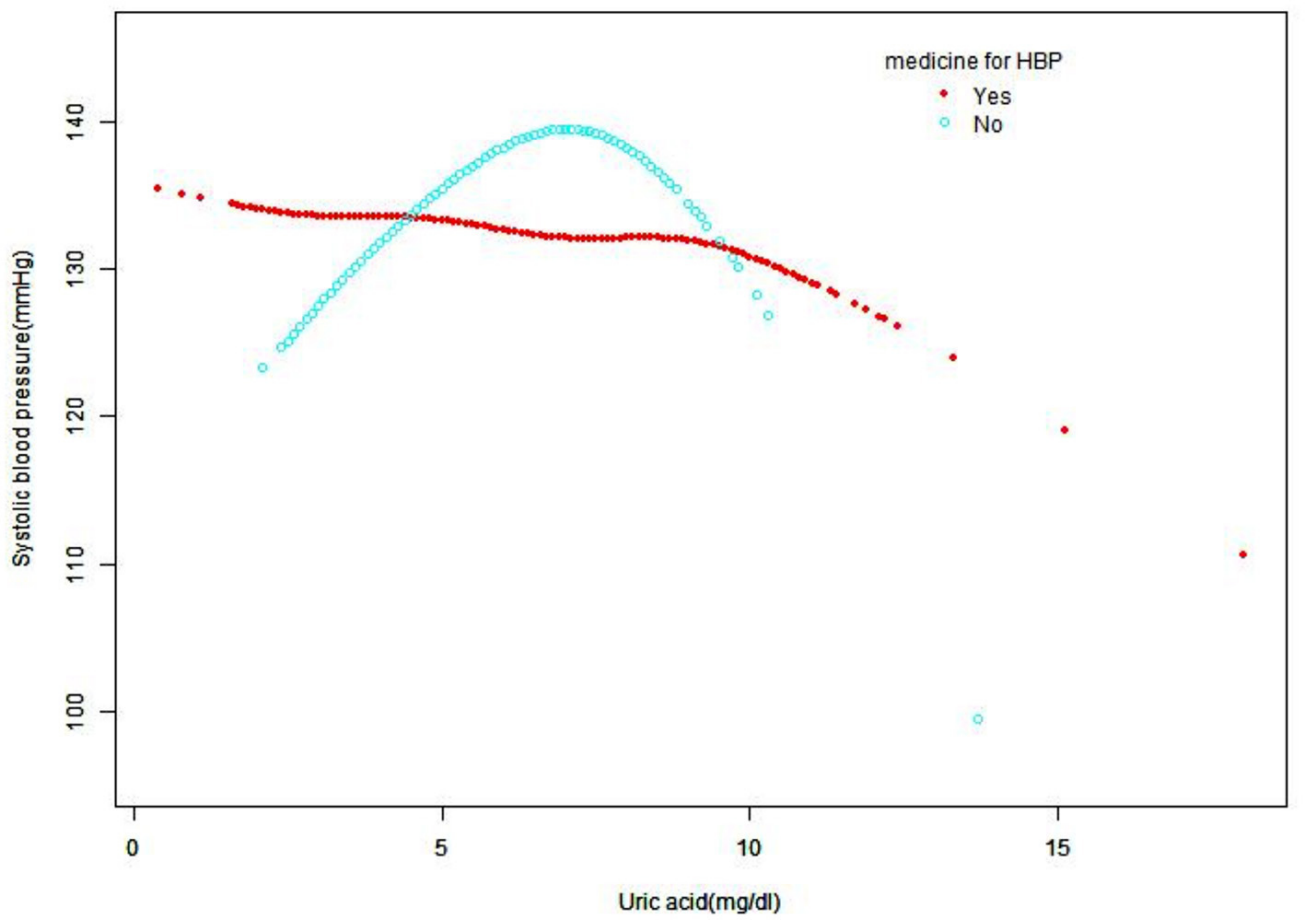
Smooth curve fitting for the relationship between UA and SBP in hypertension treatment group and non-treatment group, adjusted for gender, age, race, creatinine, glucose, hemoglobin, HDL, TC, GFR, LDL, alcohol consumption, diabetes, smoking, and BMI. HDL, high-density lipoprotein; TC, total cholesterol; GFR, glomerular filtration rate; SBP, systolic blood pressure; UA, uric acid; LDL, low-density lipoprotein; BMI, body mass index; HBP, high blood pressure.

**Figure 3:**
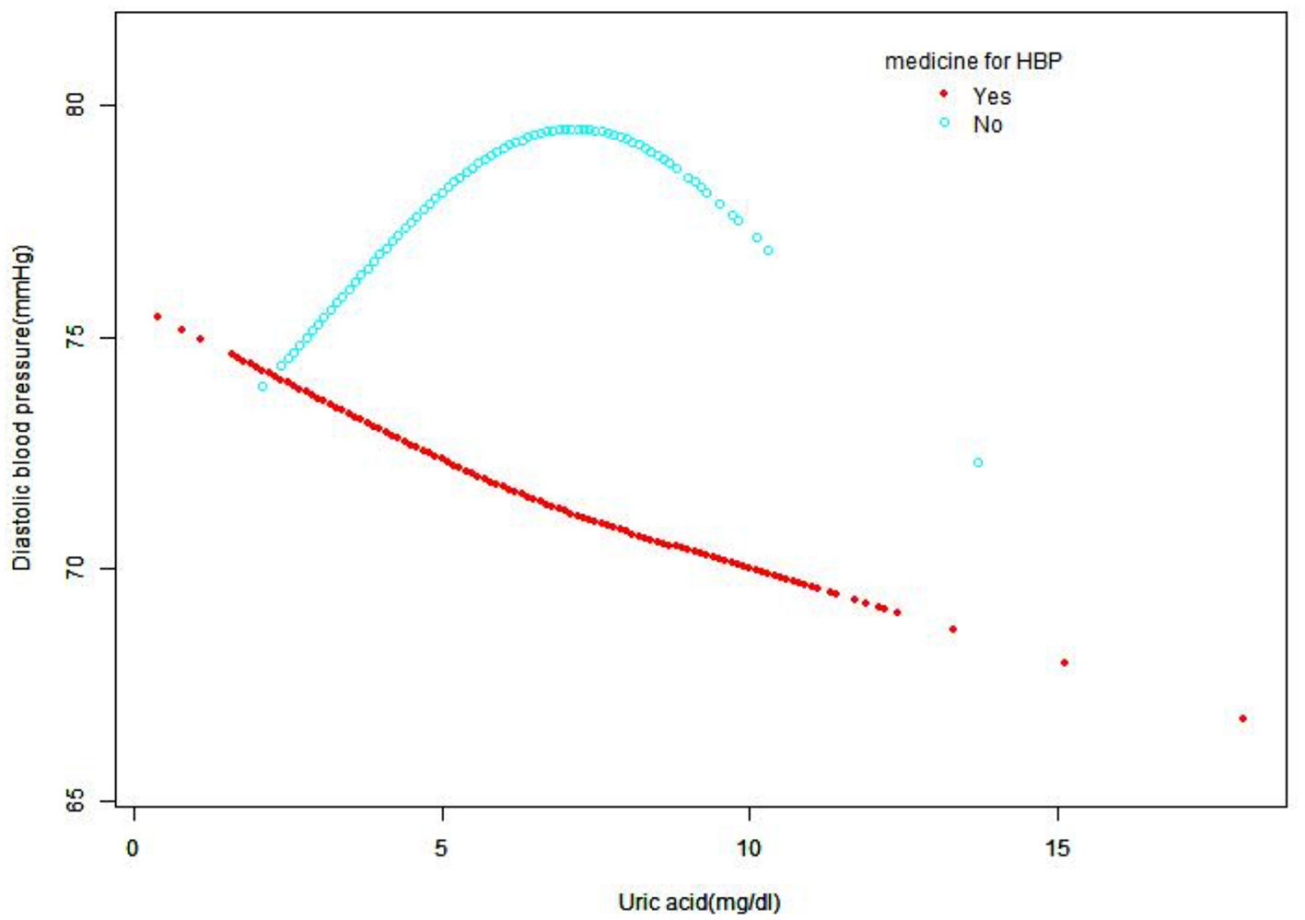
Smooth curve fitting for the relationship between UA and DBP in hypertension treatment group and non-treatment group, adjusted for gender, age, race, creatinine, glucose, hemoglobin, HDL, TC, GFR, LDL, alcohol consumption, diabetes, smoking, and BMI. HDL, high-density lipoprotein; TC, total cholesterol; GFR, Glomerular filtration rate; DBP, diastolic blood pressure; UA, uric acid; LDL, low-density lipoprotein; BMI, body mass index; HBP, high blood pressure.

A smooth curve fitting was used to explore the non-linear relationship between UA and SBP and DBP. From Figures 2, 3, the associations between UA and BP including SBP and DBP which were both a linear relationship basically were found in the hypertension treatment group. As the level of UA increased, SBP and DBP showed a gradual decrease. However, in the hypertension non-treatment group, the associations between UA and BP including SBP and DBP were both an inverted U-shape. So, a two-piece linear regression model was utilized to calculate the inflection point, and the inflection point of SBP and DBP was 7 and 7.5 mg/dl, respectively (Table 3). For SBP, the association was positively significant (β, 3.11 [95% CI, 1.67, 4.56]) before the inflection point of 7 mg/dl. However, after the inflection point of 7 mg/dl, the association was negative (β, −5.44 [95% CI, −8.60, −2.28]). For DBP, the inflection point was 7.5 mg/dl, and the effect size was positive (β, 1.19 [95% CI,0.37, 2.01]) before the inflection point. However, after it, the effect size was negative (β, −3.24 [95% CI, −5.72, −0.76]). We also conducted a smooth curve fitting by gender in the hypertension treatment group and the non-treatment group. From Supplementary Figures 1, 2, the negative association can still be seen in the hypertension treatment group stratified by gender. From Supplementary Figures 3, 4, the inverted U-shaped relationship also existed in the hypertension non-treatment group. With the exception of females, the association between UA and DBP was positive.

**Table 3:** Threshold effect analysis of UA on SBP and DBP using piecewise linear regression in the hypertension non-treatment group.

**Inflection point of UA**	**β (95% CI)**	***P*-value**
Systolic blood pressure (mmHg)
<7 mg/dl	3.11 (1.67, 4.56)	<0.0001
≥7 mg/dl	−5.44 (−8.60, −2.28)	0.0008
Diastolic blood pressure
<7.5 mg/dl	1.19 (0.37, 2.01)	0.0044
≥7.5 mg/dl	−3.24 (−5.72, −0.76)	0.0106

*CI, confidence interval; UA, uric acid; SBP, systolic blood pressure; DBP, diastolic blood pressure. Adjustment for: gender, age, race, creatinine, glucose, hemoglobin, HDL, TC, GFR, LDL, alcohol consumption, diabetes, smoking, and BMI*.

## Discussion

In our study, we found that in the hypertension treatment group, a linear relationship was identified between UA and BP including SBP and DBP. As the level of UA increased, SBP and DBP showed a gradual decrease. However, in the hypertension non-treatment group, the relationship was an inverted U-shape. So as far as we know, this is the first study to explore the association between UA and BP including SBP and DBP in hypertension with and without treatment in NHANES.

Uric acid (UA) may play an important role in the development of hypertension through the following pathophysiological mechanisms: renal afferent artery disease, renin-angiotensin-aldosterone system (RAAS) upregulation, oxidative stress, systemic inflammation, and endothelial dysfunction (11, 12). Hyperuricemia animal models and experimental studies on human cell culture have identified that UA can upregulate the RAAS (13–16). Moreover, UA-mediated inflammation can indirectly activate RAAS (15). UA has been proved to promote the occurrence of hypertension through crystal and pressure-independent kidney afferent arteriolopathy (17, 18). Conversely, many antihypertensive drugs including diuretics, RAAS antagonists, and beta blockers can increase the level of UA (19, 20). On the other hand, angiotensin II receptor antagonists and calcium channel antagonists have been proven to reduce the concentration of UA (21–23).

As mentioned before, previous studies have confirmed that UA is associated with elevated BP and the occurrence of hypertension, but the results of clinical trials are unsatisfactory. This may be a comprehensive result of the interaction between UA and antihypertensive drugs. UA can affect BP in multiple ways, and antihypertensive drugs can, in turn, affect UA levels. Another mechanism to explain this negative association may be related to hemodynamics. A decrease in BP was often accompanied by a decrease in GFR, which may lead to an increase in UA levels. In our research, we converted UA from continuous variables into categorical variables (quintiles) and found that as the level of UA increased, the GFR gradually decreased (Supplementary Table 3). Our research suggested that there was a negative correlation between UA and BP in the hypertension treatment group, which may indicate that in people with hypertension and hyperuricemia, routinely initiating uric acid-lowering therapy to reduce BP is not necessary for every patient and that individualized treatment is needed. In the hypertension non-treatment group, there was an inverted U-shaped relationship between UA and BP. When UA is within a certain level, BP can be lowered by reducing the UA level through diet or drugs. As far as we know, this was the first time that the relationship between UA and BP in a hypertension non-treatment group has been explored, and the underlying mechanism of this U-shaped curve needs to be further explored in future animal experiments and clinical studies.

However, our research has some limitations. First, some variables were based on the subjective response on the questionnaire, and there may be a certain recall bias. Second, for the hypertension treatment group, the classification of hypertension and type of antihypertensive drugs were not totally identified. Lastly, the mean blood pressure obtained by measuring blood pressure continuously for a short period of time may not fully reflect the true circumstances behind blood pressure.

## Conclusions

In our study, we found that the association between UA and BP was negative in the hypertension treatment group. In the hypertension non-treatment group, the associations between UA and BP including SBP and DBP were both an inverted U-shape, and the inflection point of SBP and DBP was 7 and 7.5 mg/dl, respectively.

## Data Availability Statement

The original contributions presented in the study are included in the article/Supplementary Material, further inquiries can be directed to the corresponding author.

## Ethics Statement

The studies involving human participants were reviewed and approved by the ethics review board of the National Center for Health Statistics approved all NHANES protocols, and written informed consents were obtained from all participants or their proxies. The patients/participants provided their written informed consent to participate in this study.

## Author Contributions

Conception and design were handled by YS and ND. Administrative support was provided by ND. Provision of study materials or patients was handled by CL, YL, and LH. Collection and assembly of data were handled by YS and ND. Data analysis and interpretation were carried out by YS and LH. Final approval of the manuscript was by all authors.

## Conflict of Interest

The authors declare that the research was conducted in the absence of any commercial or financial relationships that could be construed as a potential conflict of interest.

## Publisher's Note

All claims expressed in this article are solely those of the authors and do not necessarily represent those of their affiliated organizations, or those of the publisher, the editors and the reviewers. Any product that may be evaluated in this article, or claim that may be made by its manufacturer, is not guaranteed or endorsed by the publisher.

## Abbreviations

HDL: high-density lipoprotein
TC: total cholesterol
GFR: glomerular filtration rate
SBP: systolic blood pressure
DBP: diastolic blood pressure
UA: uric acid
LDL: low-density lipoprotein
BMI: body mass index
CI: confidence interval
CR: creatinine
GLU: glucose
HGB: hemoglobin.
