# Supplementary material for: Association of Uric Acid With Blood Pressure in Hypertension Between Treatment Group and Non-treatment Group
Source: Front Cardiovasc Med. 2022 Jan 11;8:751089. doi: 10.3389/fcvm.2021.751089 (PMC8787103; doi:10.3389/fcvm.2021.751089)
Supplement: Supplementary file 1 [file Table_1.DOCX]

**Supplementary table1. Univariate analysis for systolic blood pressure**

| Medicine for hypertension | Yes(β,95%CI,P) | No(β,95%CI,P) |
| --- | --- | --- |
| Creatinine(mg/dl) | 0.41 (-0.26, 1.09) 0.2313 | 0.75 (-1.44, 2.95) 0.5011 |
| Glucose(mmol/L) | 0.16 (-0.02, 0.33) 0.0790 | 0.44 (-0.02, 0.89) 0.0635 |
| Hemoglobin (g/dL) | -0.56 (-0.88, -0.24) 0.0006 | 0.95 (0.17, 1.74) 0.0174 |
| HDL (mmol/L) | 3.67 (2.54, 4.81) <0.0001 | 4.95 (1.77, 8.13) 0.0023 |
| TC(mmol/L) | 1.77 (1.33, 2.21) <0.0001 | 2.57 (1.34, 3.80) <0.0001 |
| GFR(ml/min/1.73m^2^) | -0.01 (-0.03, 0.01) 0.5812 | -0.11 (-0.16, -0.06) <0.0001 |
| LDL(mmol/L) | 1.65 (0.90, 2.40) <0.0001 | 3.28 (1.19, 5.37) 0.0023 |
| Gender |  |  |
| Male | Ref | Ref |
| Female | 0.86 (-0.11, 1.83) 0.0827 | -3.32 (-6.05, -0.59) 0.0175 |
| Race |  |  |
| Mexican-American | Ref | Ref |
| White | -4.85 (-6.45, -3.25) <0.0001 | -4.08 (-8.20, 0.04) 0.0527 |
| Black | 0.93 (-0.72, 2.58) 0.2715 | 3.71 (-0.63, 8.05) 0.0942 |
| Other Race | -0.53 (-2.28, 1.22) 0.5539 | -1.40 (-5.93, 3.13) 0.5446 |
| Alcohol consumption |  |  |
| No drinking | Ref | Ref |
| Drinking | -1.57 (-2.75, -0.39) 0.0090 | -0.97 (-4.69, 2.75) 0.6086 |
| Not recorded | 1.67 (0.20, 3.14) 0.0258 | -0.82 (-5.48, 3.85) 0.7312 |
| Diabetes |  |  |
| Yes | Ref | Ref |
| No | -0.95 (-2.01, 0.11) 0.0798 | 0.21 (-3.69, 4.12) 0.9147 |
| Borderline | -0.34 (-2.82, 2.14) 0.7885 | 1.85 (-5.43, 9.13) 0.6187 |
| Not recorded | 11.62 (-14.93, 38.18) 0.3909 | -10.12 (-41.02, 20.77) 0.5209 |
| Smoke |  |  |
| Smoking | Ref | Ref |
| No smoking | 0.07 (-1.37, 1.51) 0.9269 | -1.40 (-5.24, 2.43) 0.4740 |
| Not recorded | 1.47 (0.13, 2.82) 0.0317 | -0.96 (-4.17, 2.24) 0.5562 |
| Age(years) |  |  |
| 16-44 | Ref | Ref |
| 45-59 | 1.10 (-0.66, 2.86) 0.2201 | 6.46 (3.30, 9.63) <0.0001 |
| 60-79 | 6.30 (4.67, 7.94) <0.0001 | 11.38 (8.01, 14.74) <0.0001 |
| BMI (kg/m2) |  |  |
| <18.5 | Ref | Ref |
| 18.5-24.9 | -8.86 (-15.32, -2.41) 0.0071 | 2.35 (-12.49, 17.20) 0.7560 |
| 25-29.9 | -12.38 (-18.76, -6.00) 0.0001 | 5.22 (-9.47, 19.91) 0.4863 |
| ≥30 | -13.51 (-19.86, -7.16) <0.0001 | 3.89 (-10.70, 18.47) 0.6016 |
| not recorded | -9.93 (-17.55, -2.31) 0.0106 | 4.86 (-12.20, 21.92) 0.5766 |

**Abbreviations:** HDL, high-density lipoprotein; TC, total cholesterol; GFR, glomerular filtration rate; LDL, low-density lipoprotein; BMI, body mass index, Ref, reference; CI, confident interval.
