# Supplementary material for: Association of Uric Acid With Blood Pressure in Hypertension Between Treatment Group and Non-treatment Group
Source: Front Cardiovasc Med. 2022 Jan 11;8:751089. doi: 10.3389/fcvm.2021.751089 (PMC8787103; doi:10.3389/fcvm.2021.751089)
Supplement: Supplementary file 2 [file Table_2.DOCX]

**Supplementary Table2. Univariate analysis for diastolic blood pressure**

| Medicine for hypertension | Yes(β,95%CI,P) | No(β,95%CI,P) |
| --- | --- | --- |
| Creatinine(mg/dl) | -0.88 (-1.33, -0.44) <0.0001 | -0.47 (-1.85, 0.90) 0.5018 |
| Glucose(mmol/L) | -0.24 (-0.36, -0.13) <0.0001 | 0.01 (-0.28, 0.30) 0.9282 |
| Hemoglobin (g/dL) | 1.66 (1.45, 1.87) <0.0001 | 1.21 (0.72, 1.69) <0.0001 |
| HDL (mmol/L) | -0.82 (-1.57, -0.07) 0.0324 | -0.99 (-3.00, 1.01) 0.3313 |
| TC(mmol/L) | 1.80 (1.51, 2.09) <0.0001 | 1.99 (1.23, 2.76) <0.0001 |
| GFR(ml/min/1.73m^2^) | 0.05 (0.03, 0.06) <0.0001 | -0.04 (-0.08, -0.01) 0.0069 |
| LDL(mmol/L) | 2.08 (1.60, 2.57) <0.0001 | 2.37 (1.09, 3.64) 0.0003 |
| Gender |  |  |
| Male | Ref | Ref |
| Female | -2.11 (-2.75, -1.47) <0.0001 | -3.12 (-4.83, -1.41) 0.0004 |
| Race |  |  |
| Mexican-American | Ref | Ref |
| White | -0.70 (-1.76, 0.36) 0.1946 | -0.93 (-3.51, 1.66) 0.4832 |
| Black | 2.76 (1.67, 3.85) <0.0001 | 3.71 (0.99, 6.43) 0.0077 |
| Other Race | 1.58 (0.42, 2.74) 0.0074 | 1.56 (-1.28, 4.41) 0.2818 |
| Alcohol consumption |  |  |
| No drinking | Ref | Ref |
| Drinking | 2.23 (1.45, 3.00) <0.0001 | 1.41 (-0.92, 3.74) 0.2363 |
| Not recorded | 0.55 (-0.42, 1.52) 0.2636 | 0.56 (-2.36, 3.49) 0.7061 |
| Diabetes |  |  |
| Yes | Ref | Ref |
| No | 3.39 (2.70, 4.09) <0.0001 | 3.27 (0.83, 5.71) 0.0087 |
| Borderline | 3.30 (1.67, 4.93) <0.0001 | 4.28 (-0.26, 8.83) 0.0650 |
| Not recorded | -5.24 (-22.61, 12.14) 0.5546 | -13.73 (-33.02, 5.56) 0.1634 |
| Smoke |  |  |
| Smoking | Ref | Ref |
| No smoking | -2.92 (-3.87, -1.97) <0.0001 | -1.64 (-4.05, 0.76) 0.1810 |
| Not recorded | -0.41 (-1.29, 0.47) 0.3634 | -0.38 (-2.39, 1.63) 0.7130 |
| Age(years) |  |  |
| 16-44 | Ref | Ref |
| 45-59 | -3.16 (-4.27, -2.05) <0.0001 | 1.21 (-0.79, 3.21) 0.2363 |
| 60-79 | -10.22 (-11.25, -9.18) <0.0001 | -4.61 (-6.73, -2.48) <0.0001 |
| BMI (kg/m2) |  |  |
| <18.5 | Ref | Ref |
| 18.5-24.9 | 1.03 (-3.24, 5.31) 0.6352 | 4.42 (-4.84, 13.68) 0.3498 |
| 25-29.9 | 0.96 (-3.26, 5.18) 0.6558 | 7.20 (-1.97, 16.36) 0.1241 |
| ≥30 | 1.38 (-2.82, 5.59) 0.5191 | 8.43 (-0.67, 17.53) 0.0697 |
| Not recorded | 1.77 (-3.27, 6.82) 0.4911 | 9.09 (-1.55, 19.73) 0.0944 |

**Abbreviations:** HDL, high-density lipoprotein; TC, total cholesterol; GFR, glomerular filtration rate; LDL, low-density lipoprotein; BMI, body mass index, Ref, reference; CI, confident interval.
