# Supplementary material for: Association of Uric Acid With Blood Pressure in Hypertension Between Treatment Group and Non-treatment Group
Source: Front Cardiovasc Med. 2022 Jan 11;8:751089. doi: 10.3389/fcvm.2021.751089 (PMC8787103; doi:10.3389/fcvm.2021.751089)
Supplement: Supplementary file 3 [file Table_3.DOC]

| Uric acid(quintiles) | Q1 | Q2 | Q3 | Q4 | Q5 |
| --- | --- | --- | --- | --- | --- |
| **Hypertension treatment group** | | | | | |
| N | 1055 | 1251 | 1146 | 1257 | 1274 |
| GFR(ml/min/1.73m^2^) | 85.94 ± 27.72 | 77.08 ± 23.35 | 70.62 ± 21.90 | 64.82 ± 20.00 | 55.54 ± 19.51 |
| **Hypertension non-treatment group** | | | | | |
| N | 233 | 232 | 195 | 191 | 151 |
| GFR(ml/min/1.73m^2^) | 99.42 ± 32.22 | 82.60 ± 24.99 | 75.43 ± 19.60 | 72.40 ± 20.29 | 63.38 ± 19.35 |

**Supplementary table 3. Description of GFR in UA(quintiles) in hypertension treatment group and hypertension non-treatment group**

**Abbreviations:** UA, uric acid; GFR, glomerular filtration rate.
