# Supplementary material for: Association of Uric Acid With Blood Pressure in Hypertension Between Treatment Group and Non-treatment Group
Source: Front Cardiovasc Med. 2022 Jan 11;8:751089. doi: 10.3389/fcvm.2021.751089 (PMC8787103; doi:10.3389/fcvm.2021.751089)
Supplement: Supplementary file 5 [file Data_Sheet_2.doc]

**
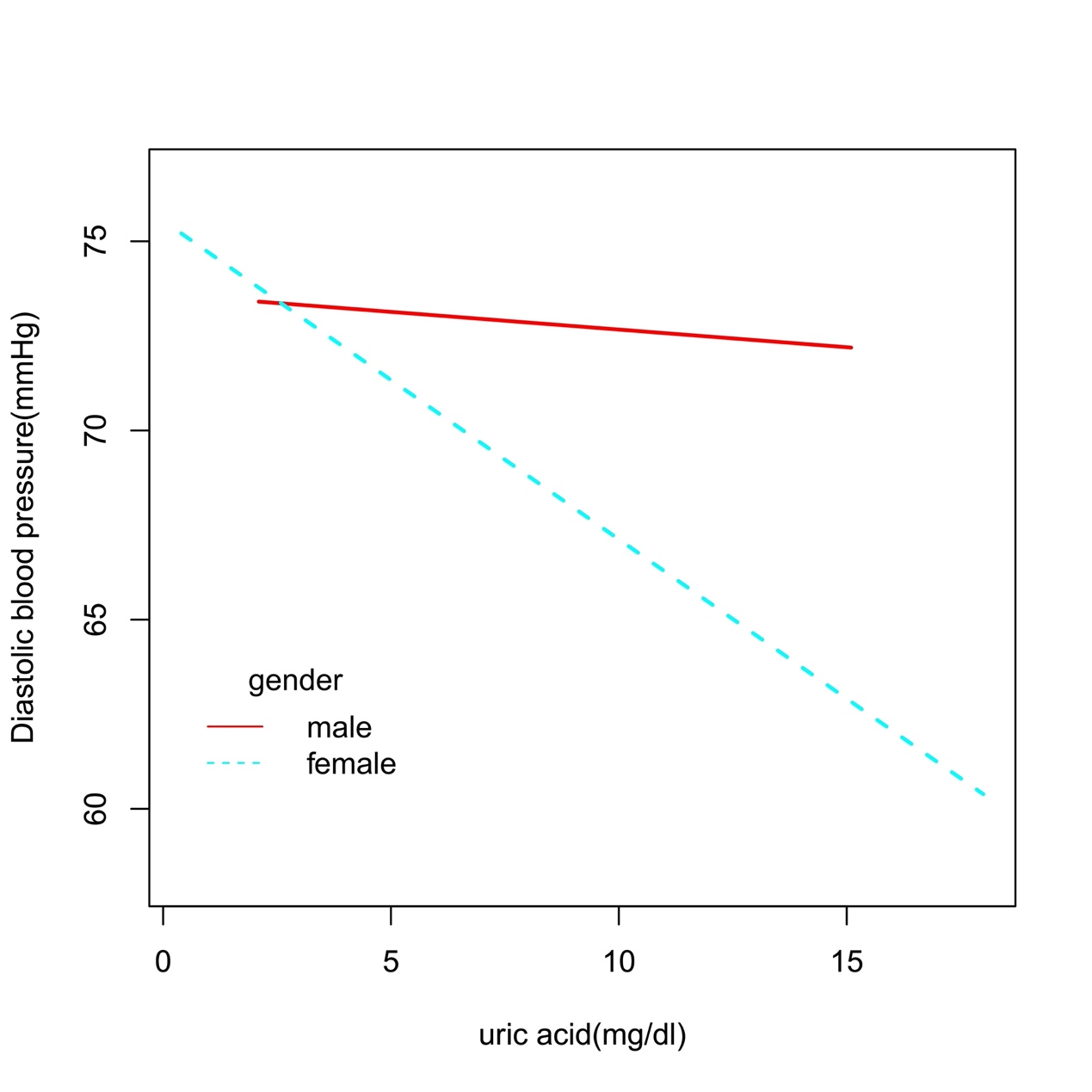
**

**Supplementary figure 2.** A smooth curve fitting for the relationship between UA and DBP in hypertension treatment group. adjust for: Age; Race; Creatinine; Glucose; Hemoglobin; HDL; TC; GFR; LDL; Alcohol consumption; Diabetes; Smoking; BMI.
